# Dysfunction of the glymphatic system in childhood absence epilepsy

**DOI:** 10.3389/fnins.2023.1312676

**Published:** 2023-12-08

**Authors:** Wanqing Pu, Shanzhen Wei, Mengdi Qiu, Xiaoyu Chen, Wenwei Zou, Yingchao Ge, Wenchao Qiu

**Affiliations:** ^1^Department of Neurology, The Fifth People’s Hospital of Huai’an, Huai’an, China; ^2^Department of Electrophysiology, The Affiliated Huai’an Hospital of Xuzhou Medical University, Huai’an, China; ^3^Department of Radiology, The Affiliated Huai’an Hospital of Xuzhou Medical University, Huai’an, China; ^4^Department of Neurology, The Affiliated Huai’an Hospital of Xuzhou Medical University, Huai’an, China; ^5^Department of Neurology, Qidong People’s Hospital, Nantong, China

**Keywords:** diffusion tensor imaging, DTI-ALPS, childhood absence epilepsy, glymphatic system, neuroimage biomarker

## Abstract

**Objective:**

This study aimed to evaluate the glymphatic system in childhood absence epilepsy (CAE) using diffusion tensor image analysis along the paravascular space (DTI-ALPS) index. Methods: Forty-two CAE patients and 50 age- and gender-matched healthy controls (HC) were included in this study. All participants underwent scanning using a Siemens 3.0 T magnetic resonance scanner, and the DTI-ALPS index was calculated. The study compared the differences of DTI-ALPS index between CAE patients and the healthy controls. Additionally, this study also assessed the relationship between the DTI-ALPS index and clinical characteristics such as age, seizure frequency, and duration of epilepsy.

**Results:**

The DTI-ALPS index was lower in CAE patients compared to the healthy controls (1.45 ± 0.36 vs. 1.66 ± 0.30, *p* < 0.01). The DTI-ALPS index showed a negative correlation with the duration of epilepsy (*r* = −0.48, *p* < 0.01) and a positive correlation with age (*r* = 0.766, *p* < 0.01) in CAE patients. However, no significant correlation was observed between the DTI-ALPS index and seizure frequency.

**Conclusion:**

The results of this study indicate that children with CAE exhibit dysfunction in the glymphatic system of the brain, which might contribute to understanding the pathophysiological mechanism of CAE. The DTI-ALPS, as a non-invasive diagnostic marker, can be used to assess the function of the glymphatic system in CAE patients, providing promising applications in the diagnosis and research of CAE.

## 1 Introduction

The glymphatic system in brain is the waste clearance system of the central nervous system (Plog and Nedergaard, 2018; Rasmussen et al., 2018). It is a glial-dependent paravascular network that allows cerebrospinal fluid (CSF) to enter the brain through the paravascular space surrounding arteries, flow into the interstitium through aquaporin-4 (AQP-4) water channels in astrocytic, and then drives interstitial fluid (ISF) and its solutes toward perivenous and perineuronal regions before eventually draining into the lymphatic vessels of the meninges and cervical lymphatics (Iliff et al., 2012; Lee et al., 2018; Mestre et al., 2018). The existence of the glymphatic system was first revealed in a mouse model using two-photon microscopy in 2012, and subsequent imaging studies have successfully visualized the glymphatic system in animal brains using radiotracer tracking (Iliff et al., 2012; Lee et al., 2018; Mestre et al., 2018). The physiological role of the glymphatic system has been extensively studied in various neurological disorders, including Alzheimer’s disease, Parkinson’s disease, traumatic brain injury, and normal pressure hydrocephalus (Iliff et al., 2012; Plog and Nedergaard, 2018; Rasmussen et al., 2018). These studies consistently indicate that dysfunction of the glymphatic system plays a significant role in the development and progression of neurological diseases. Previous investigations in humans have mainly relied on invasive methods such as intrathecal tracer injections followed by magnetic resonance imaging for studying the structure and function of the glymphatic system (Iliff et al., 2013; de Leon et al., 2017; Ringstad et al., 2017). However, the non-invasive visualization and study of glymphatic system function in the human brain has been a focus of ongoing research efforts.

A recent study proposed a method called diffusion tensor image analysis along the paravascular space (DTI-ALPS) as a non-invasive and safe imaging analysis approach to investigate the function of the brain’s glymphatic system (Taoka et al., 2017). The DTI-ALPS index evaluates the movement of water molecules in the paravascular space (PVS) direction by measuring the diffusivity. In the calculation of the DTI-ALPS index, the diffusivity along the x-axis of the projection fibers and association fibers is primarily used, and it is corrected by combining the diffusivity along the y-axis in the projection fibers and the diffusivity along the z-axis in the association fibers to evaluate the diffusivity along the paravascular space. Therefore, a decrease in the DTI-ALPS index suggests a reduction in paravascular space diffusivity, indicating dysfunction of the glymphatic system. Due to the fact that the DTI-ALPS method does not require tracer injection and exhibits good inter-rater consistency (Taoka et al., 2022), this method is highly suitable for clinical research. The feasibility of this method has also been demonstrated in recent studies of various neurological disorders (Bae et al., 2021; Zhang et al., 2021).

Childhood absence epilepsy (CAE) is the most common generalized epilepsy syndrome in children, affecting 10–17% of children with epilepsy (Loiseau et al., 1995). The onset of CAE typically occurs between the ages of 4 and 10, with a peak around 6–7 years old, and it is more prevalent in girls than boys (Waaler et al., 2000). The seizure frequency in CAE can reach several times a day. Typical clinical features include brief periods of staring, sometimes accompanied by rhythmic blinking or automatisms, lasting a few seconds, and immediately returning to the baseline level of consciousness and activity. The typical EEG findings show highly characteristic generalized (bilateral, symmetric, and synchronous) 3 Hz spike-and-wave complexes (Berg et al., 2010). CAE is generally considered a benign self-limiting epilepsy, but increasing evidence suggests that CAE patients may experience significant cognitive, behavioral, and psychiatric comorbidities (Caplan et al., 2008; Masur et al., 2013).

Previous studies have shown that the main function of the brain’s glymphatic system is to clear metabolic waste from the central nervous system, with its function being activated during sleep and in a resting state during the daytime (Rasmussen et al., 2018; Mestre et al., 2020). Research on CAE suggests that compared to the wakeful state, CAE patients exhibit a significant increase in 3 Hz spike-and-wave discharges during sleep, particularly during non-rapid eye movement sleep (Sato et al., 1973), and sleep deprivation is a common trigger for frequent daytime seizures in CAE patients (Shian and Chi, 1994). These studies suggest that CAE patients may have dysfunction in the brain’s glymphatic system.

Therefore, this study aims to evaluate the function of the brain’s glymphatic system in CAE patients by analyzing and comparing DTI-ALPS index between CAE patients and healthy controls. Furthermore, this study will also analyze the relationship between glymphatic system and clinical features in CAE patients.

## 2 Materials and methods

### 2.1 Participants

A total of 42 CAE patients and 50 age- and gender-matched healthy controls were included in this study. All participants were right-handed. Children with a clinical diagnosis of CAE were included based on the following criteria: (1) Routine video-EEG showing synchronous symmetric 3 Hz spike-and-wave discharges along with typical clinical absence seizures; (2) Normal neurological and physical examinations; (3) Normal structural imaging on a 3.0 T MRI scanner. This study was approved by the Ethics Committee of the Affiliated Huai’an Hospital of Xuzhou Medical University. Written informed consent was obtained from the guardians of all participants.

### 2.2 Magnetic resonance imaging acquisition

Image data were acquired using a Siemens 3.0 T MRI scanner. Diffusion tensor imaging (DTI) data were obtained using a single-shot echo-planar imaging sequence with the following parameters: 45 axial slices, slice thickness = 3 mm, no gap between slices, repetition time (TR) = 6,600 ms, echo time (TE) = 93 ms, acquisition matrix = 128 × 128, 30 diffusion gradient directions with a b-value of 1,000 s/mm^2^, and one acquisition with a b-value of 0 s/mm^2^.Participants are instructed to lie supine and have their heads immobilized with foam padding during the scanning process in order to minimize head movement as much as possible.

### 2.3 Image processing and calculation of DTI-ALPS index

**Figure 1:**
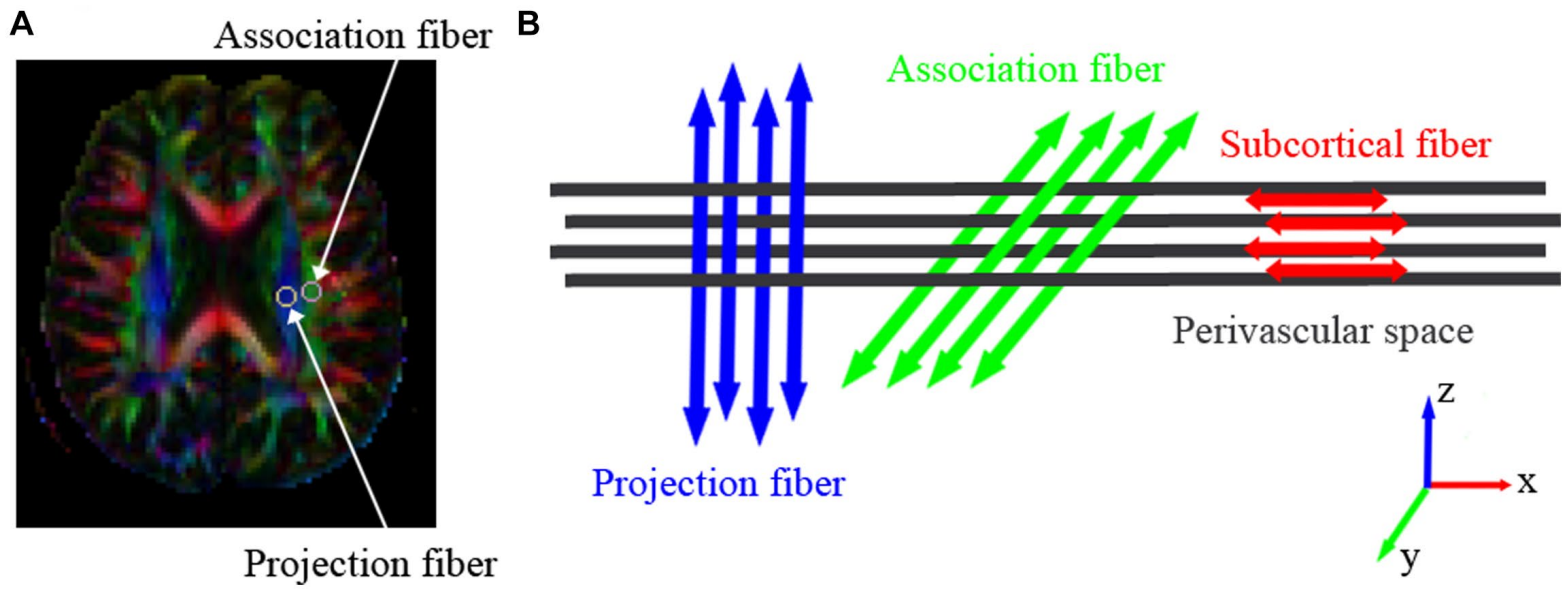
The three different neural fiber tracts at the level of the lateral ventricle body. **(A)** Colored FA maps depict the direction of the three fiber tracts: projection fibers in the Z-axis direction (blue), association fibers in the Y-axis direction (green), and subcortical fibers in the X-axis direction (red). Within the projection and association fiber tracts, a region of interest (ROI) with a diameter of 5 mm is placed to measure their diffusion coefficients. **(B)** The direction of the paravascular space (gray columns) and the orientation of the three neural fiber tracts. The direction of the paravascular space is perpendicular to the projection and association fiber tracts.

The DTI Studio^1^ was used for motion and eddy current correction and to process diffusion tensor images, including the color-coded fractional anisotropy (FA) map and the tensor (Dxx, Dyy, Dzz, Dxy, Dxz, Dzz) map. From each tensor image, diffusivity in X, Y, and Z- axis were obtained. Based on the FA map, regions of interest (ROIs) with a diameter of 5 mm were delineated in the projection fibers and association fibers of the left hemisphere (all participants were right-handed) at the level of the lateral ventricle body (Figure 1A), allowing the acquisition of diffusivity in the X, Y, and Z-axis for each type of fiber (Figure 1B). The DTI-ALPS index was calculated using the following formula:

(1) $$DTI-\mathrm{ALPS}\;\mathrm{index}=\frac{\mathrm{mean}\left(D_{\mathrm{xxproj}},D_{\mathrm{xxassoc}}\right)}{\mathrm{mean}\left(D_{\mathrm{yyproj}},D_{\mathrm{zzassoc}}\right)}$$

Where Dxxproj and Dxxassoc represent the Dxx values (diffusivity in the X-axis direction) for the projection fibers and association fibers, respectively. And Dyyproj represents the diffusivity in the Y-axis direction for the projection fibers, while Dzzassoc represents the diffusivity in the Z-axis direction for the association fibers.

### 2.4 Statistical analysis

Categorical variables were presented as percentages, and normally distributed continuous variables were expressed as mean ± standard deviation. Statistical comparisons for categorical variables were performed using the chi-square test, while independent sample t-tests were used for continuous variables. Pearson correlation coefficient was used to analyze the correlation between DTI-ALPS indices and clinical data, such as age, seizure frequency, and disease duration in CAE patients. Statistical significance was defined as a two-tailed *p*-value <0.05.

## 3 Results

The clinical characteristics of CAE patients and the comparison results of demographic data with healthy controls are detailed in Table 1. There were no significant differences in age (8.31 ± 2.12 years vs. 8.44 ± 1.94 years, *p* = 0.76) and gender (males, 22 [52.38%] vs. 28 [56.00%], *p* = 0.40) between the CAE and healthy controls.

**Table 1:** Baseline characteristics for participants.

	CAE (*n* = 42)	HC (*n* = 50)	*P*-value
Age (years, mean ± SD)	8.31 ± 2.12	8.44 ± 1.94	0.76
Gender (F/M)	20/22	22/28	0.40
Disease duration (months, mean ± SD)	10.71 ± 4.97	NA	–
Seizure frequency (times/day, mean ± SD)	7.71 ± 2.00	NA	–

CAE, childhood absence epilepsy; HC, heathy control; M, male; F, female; SD, standard deviation; NA, not applicable.

**Figure 2:**
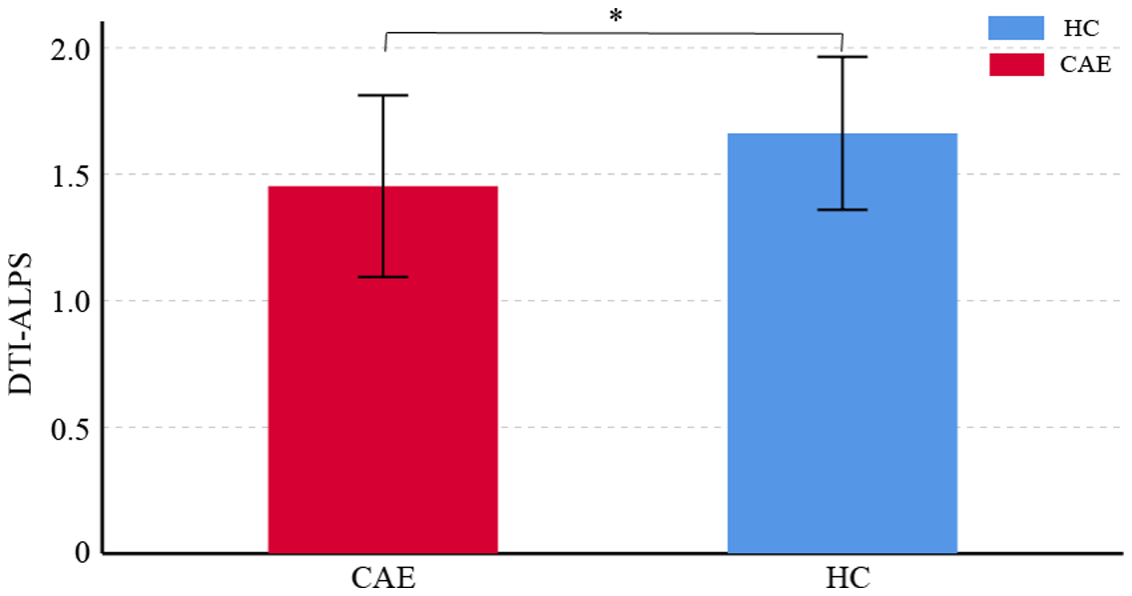
Comparisons of DTI-ALPS index between CAE patients and healthy controls (HC). The asterisk shows significant between-group difference (*p < 0.01). The error bar indicates standard deviation.

Compared to the healthy control group, CAE patients showed a significant reduction in DTI-ALPS index, and the difference between the two groups was statistically significant (1.45 ± 0.36 vs. 1.66 ± 0.30, *p* < 0.01) (see Figure 2, for details of the diffusivity along x/y/z-axis, please see Supplementary Table S1).

**Figure 3:**
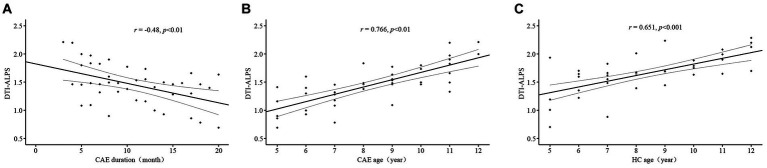
Scatter plots of the correlation analysis. **(A)** Significant negative correlation between DTI-ALPS index and the duration of epilepsy in the CAE group. **(B)** Positive correlation between DTI-ALPS index and age in the CAE group. **(C)** Positive correlation between DTI-ALPS index and age in the HC group.

The results of the correlation analysis showed a negative correlation between DTI-ALPS index and disease duration in CAE patients (*r* = −0.48, *p* < 0.01, Figure 3A), and a positive correlation between DTI-ALPS index and age (*r* = 0.766, *p* < 0.01, Figure 3B). However, no significant correlation was found between DTI-ALPS index and seizure frequency.

In the healthy controls, DTI-ALPS index was also significantly positively correlated with age (*r* = 0.651, *p* < 0.001, Figure 3C).

## 4 Discussion

In this study, we employed the DTI-ALPS index to investigate the function of the brain glymphatic system in patients with CAE compared to a healthy control group. The main findings of this study revealed that the DTI-ALPS index in CAE patients was significantly lower than that in the healthy controls, indicating impaired function of the brain glymphatic system in CAE patients. Furthermore, a negative correlation was observed between the DTI-ALPS index and the duration of epilepsy, suggesting a gradual decline in the function of brain glymphatic system in CAE patients with prolonged disease course.

With the growing attention to the glymphatic system, numerous studies have reported on the relationship between neurodegenerative diseases and the glymphatic system. It is well known that reduced clearance of amyloid-β plays a role in the pathophysiology of Alzheimer’s disease (AD) (Kress et al., 2014), and decreased clearance of phosphorylated tau is associated with chronic traumatic encephalopathy (Plog et al., 2015). Similarly, impaired clearance of metabolic byproducts in the interstitial fluid and disrupted circulation between cerebrospinal fluid and interstitial fluid have been observed in epilepsy (Marchi et al., 2016). Studies have shown that epileptic seizures lead to compromised blood–brain barrier integrity, allowing the transfer and accumulation of certain solutes from the serum into the brain parenchyma, and alterations in the ion composition in the interstitial fluid, contributing to the occurrence of brain edema (Oby and Janigro, 2006). Prolonged pathological conditions may alter the flow of interstitial fluid in the brain, resulting in a decline in clearance system functionality and accumulation of toxic molecules in the brain.

Previous studies have reported the application of the DTI-ALPS index to assess the functionality of the brain glymphatic system in neurodegenerative diseases. A study focusing on patients with Alzheimer’s disease demonstrated a significant correlation between the DTI-ALPS index and disease severity (Taoka et al., 2017). In a comparative study between patients with Parkinson’s disease and essential tremor, it was found that the DTI-ALPS index was significantly lower in the Parkinson’s disease group compared to essential tremor patients (McKnight et al., 2021). In a study investigating normal pressure hydrocephalus, it was observed that patients with normal pressure hydrocephalus had a significantly lower DTI-ALPS index compared to the healthy control group (Bae et al., 2021).

Previous studies on DTI-ALPS and epilepsy have shown that various types of epilepsy, such as juvenile myoclonic epilepsy, status epilepticus, newly diagnosed focal epilepsy, and temporal lobe epilepsy, are associated with impaired functionality of the brain glymphatic system (Lee H.J. et al., 2022; Lee et al., 2022a,b; Kim J. et al., 2023). The findings of this study indicate that children with absence epilepsy also exhibit impaired functionality of the brain glymphatic system. Therefore, we hypothesize that the dysfunction of the brain glymphatic system may not be limited to specific types of epilepsy but rather a common pathophysiological phenomenon in all epilepsy patients. Unfortunately, the exact reason underlying the glymphatic system dysfunction in patients with epilepsy is unclear. Many reports have focused on this topic and several factors have been explored such as inflammation (Gorter et al., 2015), poor sleep quality (Reddy and van der Werf, 2020), impaired aquaporin 4 water channel (Lohela et al., 2022), glutamate overproduction and glutamate receptor overactivation after epileptic seizures (Gorter et al., 2015; Dadas et al., 2016). Previous neuropathological studies on epilepsy patients have demonstrated an increased number of activated microglial cells (Altmann et al., 2022), and considering that the brain glymphatic system consists of neural glial cells, this further supports our hypothesis. Nonetheless, this viewpoint requires further support from future clinical and basic research evidence.

Due to dysfunction in the glymphatic system, misfolded proteins in many neurological disorders accumulate both intracellularly and extracellularly, leading to abnormal distribution of growth factors, neurotransmitters, carrier proteins, and other solutes. The results of this study indicate a negative correlation between the DTI-ALPS index and disease duration in patients with CAE, suggesting a progressive worsening of glymphatic system dysfunction with prolonged disease course. Building upon previous research, we speculate that this phenomenon may be related to the impact of epileptic seizures on the integrity of the blood–brain barrier (Marchi et al., 2016). Epileptic seizures can potentially compromise the integrity of the blood–brain barrier, allowing substances from the serum to permeate and accumulate in the brain parenchyma. This may interfere with the normal functioning of the glymphatic system in the brain, leading to impaired waste clearance. However, the specific mechanisms and consequences require further investigation for a more comprehensive understanding.

A previous study concerning children with autism spectrum disorder reported a positive correlation between age and ALPS-index (Li et al., 2022). Consistently, we also observed a significant positive correlation between the DTI-ALPS index and age in both CAE patients and healthy controls. We believe that this finding may be attributed to the inclusion of pediatric subjects in this study who are in the midst of growth and development. However, beyond the age of 40 in adults, a negative correlation is observed (McKnight et al., 2021; Lee et al., 2022a,b), as the number of polarized aquaporin-4 channels on the end feet of astrocyte cells and CSF production decrease along with arterial pulsatility (Kress et al., 2014; Jessen et al., 2015).

Although this study yielded meaningful results, there are still limitations to consider. Firstly, the sample size was relatively small and some of the CAE patients was taking one or two anti-seizure medications which could have an effect on the DTI-ALPS index (Kim S.T. et al., 2023). Future studies with larger sample sizes are needed to further validate our findings and a sub-group analysis based on different anti-seizure medications will be preferred. Secondly, as a cross-sectional study, while we observed an association between abnormal brain glymphatic system function and disease duration in CAE patients, causality between the two cannot be determined. Future prospective studies with larger sample sizes are warranted to investigate the causal relationship between seizure activity and dysfunction of the brain glymphatic system. Additionally, although we used FA maps to identify fiber orientations, the selection of fiber tracts in three directions and measurement of diffusivity were done manually by drawing regions of interest (ROIs), which may introduce inter-rater variability.

In conclusion, this study indicates dysfunction of the brain glymphatic system in patients with CAE. Furthermore, the functionality of the glymphatic system is closely associated with age and disease duration. Therefore, our findings suggest that the DTI-ALPS index can reflect the brain glymphatic system function in CAE patients and may serve as a potential imaging biomarker for diagnosing and monitoring the functionality of the brain glymphatic system in epilepsy patients.

## Data availability statement

The raw data supporting the conclusions of this article will be made available by the authors, without undue reservation.

## Ethics statement

The studies involving humans were approved by the Ethics Committee of the Affiliated Huai’an Hospital of Xuzhou Medical University. The studies were conducted in accordance with the local legislation and institutional requirements. Written informed consent for participation in this study was provided by the participants’ legal guardians/next of kin.

## Author contributions

WP: Methodology, Resources, Writing – original draft. SW: Data curation, Writing – original draft. MQ: Data curation, Methodology, Writing – original draft. XC: Resources, Writing – original draft. WZ: Data curation, Resources, Writing – original draft. YG: Funding acquisition, Project administration, Writing – review & editing. WQ: Conceptualization, Funding acquisition, Project administration, Writing – review & editing.

## Footnotes

^1^ https://www.mristudio.org/
