# Supplementary material for: Dysfunction of the glymphatic system in childhood absence epilepsy
Source: Front Neurosci. 2023 Dec 8;17:1312676. doi: 10.3389/fnins.2023.1312676 (PMC10749194; doi:10.3389/fnins.2023.1312676)
Supplement: Supplementary file 1 [file Table_1.DOCX]

Supplementary Material

| **Supplementary Table 1. The differences in diffusivities between patients with CAE and healthy controls** | | | |
| --- | --- | --- | --- |
|  | **CAE (n=42)** | **HC (n=50)** | ***P***-value |
| Projection fiber |  |  |  |
| Dxx | 0.00056±0.0001 | 0.00059±0.00009 | 0.058 |
| Dyy | 0.00045±0.0001 | 0.00042±0.0001 | 0.063 |
| Dzz | 0.00107±0.00011 | 0.00105±0.0001 | 0.287 |
| Association fiber |  |  |  |
| Dxx | 0.00062±0.00012 | 0.00065±0.00011 | 0.411 |
| Dyy | 0.00114±0.00012 | 0.00115±0.00014 | 0.662 |
| Dzz | 0.00036±0.00011 | 0.00033±0.00008 | 0.057 |
| Subcortical fiber |  |  |  |
| Dxx | 0.00107±0.00016 | 0.00112±0.00015 | 0.562 |
| Dyy | 0.00066±0.00018 | 0.00064±0.00018 | 0.402 |
| Dzz | 0.00058±0.00018 | 0.00062±0.00019 | 0.077 |
| CAE, childhood absence epilepsy; HC, heathy control; Dxx, diffusivity along the x-axis; Dyy, diffusivity along the y-axis; Dzz, diffusivity along the z-axis. | | | |
